# Health enhancing strength training in nonagenarians (STRONG): rationale, design and methods

**DOI:** 10.1186/1471-2458-9-152

**Published:** 2009-05-26

**Authors:** José A Serra Rexach, Jonatan R Ruiz, Natalia Bustamante-Ara, Margarita Hierro Villarán, Pedro González Gil, Maria J Sanz Ibáñez, Nekane Blanco Sanz, Victor Ortega Santamaría, Natalia Gutiérrez Sanz, Ana B Marín Prada, Cristian Gallardo, Gabriel Rodríguez Romo, Alejandro Lucia

**Affiliations:** 1 Geriatric Department, Hospital General Universitario Gregorio Marañón, Madrid, Spain; 2 Department of Biosciences and Nutrition at NOVUM, Unit for Preventive Nutrition, Karolinska Institutet, Huddinge, Sweden; 3 Department of Physiology, Universidad Europea de Madrid, Madrid, Spain; 4 Medical department, Residencia Los Nogales-Pacífico, Madrid, Spain; 5 Sports department, Instituto Nacional de Educación Física, Universidad Politécnica, Madrid, Spain

## Abstract

**Background:**

The Health Enhancing Strength Training in Nonagenarians (STRONG) is a randomised control trial to assess the effectiveness of an aerobic and strength training program for improving muscle strength, functional capacity and quality of life in nonagenarians.

**Methods:**

Sixty (51 women) nonagenarians (age range: 90–102 years) who live in a geriatric nursing home will be randomly assigned to either a usual care (control) group (n = 30) or an intervention (training) group (n = 30). Participants allocated in the usual care group will receive general physical activity guidelines and participants allocated in the intervention group will also enrol in three weekly non-consecutive individualized training sessions (~45–50 min each) during 8 weeks. The exercise program will consist of muscular strength [with a special focus on leg press at 30% (start of the program) to 70% 1 repetition maximum (end)] and aerobic exercises (cycle-ergometry during 3–5 to 15 minutes at 12–14 points in the rate of perceived exertion scale).

**Results:**

Results from STRONG will help to better understand the potential of regular physical activity for improving the well-being of the oldest population groups.

**Conclusion:**

The increase in life expectancy together with the dramatic decrease in birth rates in industrialized countries calls the attention to health care systems and public health policymakers to focus attention on promoting healthy lifestyle in the highest sector of the population pyramid. Our study attempts to improve functional capacity and QOL of nonagenarians by implementing an individualised aerobic and strength training program in a geriatric residential care. Results from STRONG will help to better understand the potential of regular physical activity for improving the well being even in persons aged 90 years or over.

**Trail Registration:**

ClinicalTrials.gov ID: NCT00848978

## Background

The fact that in western societies people are living longer is demanding the exploration of new roads to promote healthy ageing instead of merely treating the diseases of old age [1]. A powerful intervention for both promoting healthy ageing and treating age-related disorders is regular physical activity (PA). The American College of Sports Medicine (ACSM) and the American Heart Association (AHA) recently launched the PA recommendations for older adults (≥ 65 years): this population group should enrol in aerobic and strength activities most days of the week [2].

Maintenance of adequate muscle mass and strength plays a key role in the prevention of numerous chronic diseases and in the ability to cope with activities of daily living (ADLs) [3,4]. Resistance (strength) exercise training increases muscle mass and strength [5,6], and is currently recommended by the major health organizations for improving health and fitness [6-10]. In elderly people, functional capacity becomes more directly dependent on muscular fitness as these persons also experience age-associated declines in muscle mass (i.e. sarcopenia) and thus in muscular strength. Sarcopenia contributes to the decreased capacity for independent living and reduced ability to cope with ADLs and thus increases the burden for the caregiver and community [11]. Already in the 80s, Bortz [12] indicated that many biological changes that are commonly attributed to ageing, e.g. sarcopenia, can be reverted, since they are mostly caused by disuse.

Several prospective studies indicated that cardiorespiratory fitness and muscular strength are inversely associated with all-cause mortality [13-32]. A recent meta-analysis located 66 randomised trials on resistance exercise training interventions for older adults (mean age of 60 years and over); it was concluded that progressive strength training is effective to increase muscular strength in this subpopulation [33]. Though strength training is also effective in the eldest (86–96 years) [34], whether this intervention does also improve the functional capacity, quality of life (QOL) and ability to perform ADLs in nonagenarians (≥ 90 years) remains to be elucidated. Intervention studies examining with nonagenarians are scarce owing to the difficult access to such particular population. According to the United Nations (average for the 2005–2010 period), Spain is the sixth country in the world with the longest life expectancy at birth [35]. Therefore, especially in our country, it is of public health and clinical relevance to better understand the effects of regular PA in very old people (≥ 90 years).

### Objectives

The primary objectives of the Health Enhancing Strength Training in Nonagenarians (STRONG) trial is to assess the effectiveness of an 8-week aerobic and strength training program for improving muscle strength, daily functional capacity and quality of life (QOL) in nonagenarians. Primary outcomes were measures of muscular strength, daily physical functioning (Tinetti scale, Barthel index, ambulation ability), and QOL and well-being. We hypothesise that an individualised training program (intervention) focusing on strength exercises and delivered to nonagenarians by specialists in exercise training and health educators in a geriatric nursing home will result in a improvement in the aforementioned outcomes compared to the usual care. A secondary objective is to assess the effects of the intervention on PA levels and body composition. As such, measures of these two variables were secondary outcomes.

## Methods/Design

### Study design

The present study is a randomised controlled trial (RCT). The Medical Ethics Committee of *Hospital General Universitario Gregorio Marañón *(Madrid, Spain) approved the study design, study protocols and informed consent procedure. All participants have to provide a written informed consent. After baseline measurements, they will be randomly allocated to the control or intervention group. The participants will be followed for 8 weeks. All follow-up examinations will be performed in the same setting (geriatric nursing home, *Los Nogales-Pacífico*, Madrid, Spain) and by the same investigators as in the baseline measurements. The study will be performed between March 2009 and September 2009, following the ethical guidelines of the Declaration of Helsinki, last modified in 2000.

### Study participants and selection criteria

STRONG participants include 60 community-dwelling elderly people aged 90 years or over recruited from a geriatric nursing home (Los Nogales-Pacífico, Madrid, Spain). All participants received a comprehensive medical examination.

The inclusion criteria for STRONG are:

- Age: 90 years or over.

- Planning to stay in the same nursing home during the study.

- Able to ambulate, with or without assistance.

- Able to communicate.

- Informed consent: Must be capable and willing to provide consent.

The exclusion criteria for STRONG are:

- Acute or terminal illness.

- Myocardial infarction in the past 3 months.

- Not capable to ambulate.

- Unstable cardiovascular disease or other medical condition.

- Upper or lower extremity fracture in the past 3 months.

- Severe dementia.

- Unwillingness to either complete the study requirements or to be randomised into control or training group.

- Presence of neuromuscular disease or drugs affecting neuromuscular function.

**Figure 1:**
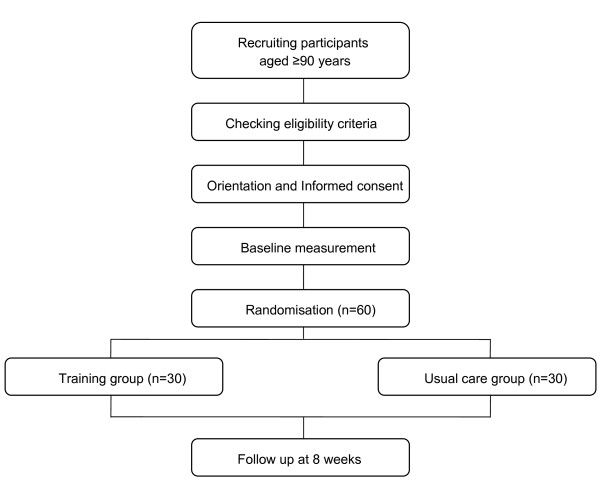
**Flow diagram of the study participants**.

Figure 1 illustrates the participant flow from recruitment to randomisation.

### Randomisation and blinding

Participants will be randomly assigned to either the control or training group with a block on gender and ambulation ability based on the Functional Ambulation Classification (FAC) scale (score 0–3 *vs *4–5) [36] by the data manager based on a computer-generated randomisation sequence. The group assignment coding (0 for usual care and 1 for intervention) will be concealed to the research group. The assessment staff will be blinded to participant randomisation assignment. Participants will be explicitly informed and reminded to not to discuss their randomisation assignment with assessment staff. It will not be possible to conceal the group assignment from the staff involved in the training.

### Sample size and statistical power

The required sample size was determined for one of the primary outcome variables, i.e. functional capacity using the Tinetti scale [37]. We believe that a clinically relevant change is a ≥ 30% increase in the aforementioned scale. We expect the control group to improve ~0 to 5%; thus, we can detect differences of at least ≥ 35% with a power > 80% and an α of 0.05 with two groups of 25 subjects. Assuming a maximum loss of follow-up of 20%, we will recruit a total of 60 nonagenarians. We will be also able to detect a significant difference of 4 points in the Tinetti scale with this number of participants, assuming a standard deviation of 5 points.

### Statistical analysis

For group comparisons at baseline (usual care *vs *intervention), we will analyse continuous variables with a Student's *t *test (or its non-parametric equivalent) for unpaired data, and Chi-square tests for nominal data. We will adjust multiple comparisons for mass significance [38,39]. We will also examine the differences between drop-outs and participants who remained in the study. We will analyse the data according to the intention-to-treat principle [40]. We will handle missing data due to drop-outs or non-compliance using multiple imputation methods. To fully appreciate the potential influence of missing responses, we will perform sensitivity analysis.

We will use a two-factor (group and time) analysis of variance (ANOVA) with repeated measures to assess the training effects on the primary outcomes [muscular strength, daily physical functioning (Tinetti scale, Barthel index, ambulation ability) and QOL and well-being] and secondary study outcomes (anthropometry and PA levels). For each outcome variable we will report the effect size and the level of significance corresponding to the main group (between-subjects), time (within-subjects) and interaction (group × time) effects. In order to prevent type I error, we will perform post hoc comparisons (pre *vs *post by group) only when a significant interaction effect is present. The level of significance will be set to = 0.05.

### Usual care group (control)

Participants randomly assigned to the usual care group will follow the general advice from the physiotherapists about the positive effects of PA. They will perform 40–45 min/day, 5 days a week, of small active and passive movements applied as a series of gentle stretches in a smooth, rhythmic fashion to the individual joints. They will also perform aerobic activities such as walking for 5–10 minutes at low intensity-intensity exercise. Intensity [expressed as rate of perceived exertion (RPE)] will range from 9 to 11 in the Borg's conventional (6–20 point) scale [41]. These RPE values correspond to a subjective perceived exertion of "very light – gentle walking" and "fairly light" respectively.

### Intervention (training)

Participants allocated in the intervention group will be enrolled in three weekly non-consecutive training sessions for 8 weeks. Each session will last for about 45–50 min. The exercise program will consist of muscular strength and aerobic exercises. Each session will start and end with a low intensity ~5–7 min warm-up and cool-down period respectively, consisting mainly of stretching exercises involving all major muscle groups.

The core portion of the training session will consist of strength training engaging the major lower limb muscles, i.e. leg press exercise performed with variable resistance weight machines (Technogym, Barcelona, Spain). The participants will perform one set of 8–10 repetitions with resting periods of 1–2 min between exercises. The load will be gradually increased as the strength of each person improves, i.e. from 30% of 1 repetition maximum (1RM) at the start of the program to 70% of 1RM at the end. Resistance training will also include one set of 8–10 repetitions of biceps curls, arm extensions, arm side lifts, shoulder elevations, seated bench press and seated lateral row. For these exercises, we will use barbells (1–3 kg per exercise) or low-to-medium resistance bands (Therabands). We will also include handgrip exercises with foam balls (3 repetitions of 10 seconds each). Stretching exercises of the muscles involved in the previous exercises will be performed during the rest periods. Participants will be given advice to complete all movements in a slow, controlled fashion, and to not to hold their breath during the exercises.

Aerobic training will be executed in a cycle ergometer (Bike Excite Forma, Technogym, Barcelona, Spain) and will include ~5 minutes (at the start of the program) to ~15 minutes of moderate-intensity exercise. Intensity (expressed as RPE) will range from 12 to 13 in the Borg's conventional (6–20 point) scale [41]. These RPE values correspond to a subjective perceived exertion of "light" and "somewhat hard" respectively. Overexertion will be checked by the "Talk test" [42]. All sessions will be performed in the exercise training facilities from the geriatric nursing home *Los Nogales-Pacífico *(Madrid, Spain).

Participants in the training group will also join to the activities performed by the usual care group two days per week.

### Participant retention and adherence

To reduce participants drop out and to maintain adherence to the training program, all sessions will be accompanied with music [43], and will be performed on an airy, well lighted exercise room. Qualified fitness specialists will carefully supervise every training session and will work with groups of 2–3 persons to ensure that participants are performing the exercises correctly.

### Primary outcome measures

#### Quality of life (QOL) and well-being

We will assess participants' QOL with the Spanish version of the Short Form-12 items (SF-12), which has shown its validity in the Spanish population [44]. We will also assess a life satisfaction index with the EuroQol instrument [45], and depressive symptoms by the geriatric depression scale [46].

#### Daily physical functioning

We will assess participants' balance and walk abilities using the *Tinetti scale *[37]. This is a simple, easily administered test that measures a patients' gait and balance. It uses a three-point ordinal scale, ranging from 0–2 where "0" indicates the highest level of impairment and "2" the individual's independence. Interpretation of scores is provided as low, medium, or high fall-risk. For balance evaluation, the subject is seated in a hard, armless chair and the following manoeuvres are tested: sitting balance, arises, attempts to arise, immediate standing balance (first 5 seconds), standing balance, 'nudged', eyes closed, turning 360 degrees and sitting down. The maximum sum-score of both gait and balance components is 28 points. Patients who score below 24 are at risk for falls, and the risk of falling is high with a score below 19. The validity of this test for screening old adults at risk for falling is well established [47].

The *Barthel index *is a valid instrument that is widely used to measure the capacity of a person for the execution of ten basic activities in daily life, obtaining a quantitative estimation of the subject's level of independency [48,49]. The ten items include: eating, transferring from bed to chair, using the toilet, bathing/showering, personal hygiene (tooth brushing, shaving) dressing, walking, stair climbing, and bowel and bladder control. Each individual item is scored with 0 (unable to perform without complete help or fecal/urine incontinence), 5 (able to perform the activity with little help or only accidental fecal/urine incontinence) or 10 (able to perform without any help or total fecal/urine continency). The sum-score ranges from 0 (*totally dependent*) to 100 (*totally independent*).

#### Muscular strength

Dynamic muscular strength of the lower body will be assessed following a standardized strength testing protocol, i.e. 1RM seated leg press, using the aforementioned variable resistance weight machines. The 1RM leg press test is a valid means to assess leg muscle strength in elderly men and women [50]. Initial loads will be 70–100% of body weight. Following a brief rest period, increments of 2–4 kg will be added until maximal effort is achieved for each lift, usually after 5 trials or less. All participants should be able to lift the initial load at least one time. Participants will be instructed on proper breathing and lifting form for each movement.

Upper body strength will be assessed with the handgrip strength test [51]. This test is a valid means for assessing upper body strength in the elderly [22,51]. Handgrip strength will be measured using a digital dynamometer (T.K.K. 5101 Grip-D; Takey, Tokyo, Japan), and the scores will be recorded in kilograms (0.1 kg). When performing the measurement, participants will be instructed to maintain the standard bipedal position during the entire test with the arm in complete extension and will not be allowed to touch any part of the body with the dynamometer except the hand being measured. Each subject will perform (alternately with both hands) the test twice, and allowing a 30–60 seconds rest between the measurements. For each measure, the hand to be tested first will be chosen randomly. The grip span of the dynamometer will be adjusted to the individual's hand size [51].

#### Ambulation ability

We will use a (i) 8-meter walk test and (ii) 4-step (20-cm height each) stairs test, both of which have proven useful to determine leg extension power and functional mobility in the elderly [52]. All the participants will use hand railing while ascending and descending the stairs to diminish the risk of falling. Performance time in all the tests will be measured by the same investigator with the same stopwatch to the nearest 0.1 s.

### Secondary outcome measures

#### Anthropometry

Standing height will be measured to the nearest 0.1 cm with a clinical stadiometer (Asimed T2, Barcelona, Spain) while the person is standing barefoot. Body mass will be determined to the nearest 0.05 kg using a balance scale (Ano Sayol S.L., Barcelona, Spain) with the person in her/his underwear. Body mass index (BMI) will be calculated as weight/height (kg/m^2^). Skinfold thickness will be measured with a Harpenden caliper at biceps, triceps, subscapular, abdominal, and suprailiac area at the left side of the body according to the criteria described by Lohman et al. [53]. We will estimate lean and fat mass according to Durnin et al. [54]. Waist circumference will be measured level with the umbilicus. These anthropometric measures are valid to assess body composition in the elderly [55]. To ensure a good reliability, all anthropometric measurements will be performed in triplicate by the same experienced researcher.

#### Physical activity (PA)

We will assess PA with a uni-axial accelerometer (Actigraph MTI, model GT1M, Manufacturing Technology Inc., Fort Walton Beach, FL, USA) which is a valid and reliable tool to asses physical activity [56,57]. Technical specifications and performance properties of this instrument have been described elsewhere [56,57]. A 60 seconds epoch will be used in this study. Participants will be instructed to place the monitor at the lower back, using an elastic waist band and wear it for seven consecutive days. They will be also instructed to wear the accelerometer during all time awake and only to remove it during water based activities.

A measure of total volume of activity (so called average intensity of PA) will be expressed as the sum of recorded counts per epoch divided by total daily registered time. We will also obtain the following measures: time spent inactive, total time in light, moderate, and vigorous intensity PA. We will calculate the time spent in at least moderate PA (so called 'moderare-to-vigorous PA' or 'MVPA'). Inactivity will be classified as activity below an arbitrary level of 100 counts per minute, including sporadic zero values less than 20 continuous minutes [58]. The cutoff points for light, moderate and vigorous PA will be set to 100-1951, 1952-5724 and ≥ 5725 counts per minute respectively [56]. A measure of total activity will be expressed as average intensity from the activity monitor and determined as the total counts per day divided by registered time (counts per minute).

We will also assess Cognitive impairment through the Mini-Mental State Examination (MMSE) [59].

### Familiarization and reliability assessment

Before the start of the study all subjects will have a familiarization period with all the tests, consisting of three ~30-min sessions. Each session will be preceded by a warm-up and will end with a cool-down of the same activities and duration used during the training period. Each familiarization session will consist of 2–3 sets of 1–3 repetitions of the exercises. We will also assess test-retest reliability for each outcome measure.

### Assessment of side effects

We will also ascertain adverse events, including muscle pain, fatigue, and general aches and pains by self-report during the study period. We will also record the falls over the study period and 1 week after. The mean incidence of falls in nursing homes is 1.5 falls per bed per year (range 0.2–3.6). Fall rates in residential care is 1.5 falls per bed per year (range0.2–3.6) and it certainly depends on the state of fragility of the person [60]. The most successful strategies for fall prevention include interventions to improve strength and functional status, reduce environmental hazards, and monitoring of high-risk residents [60]. In our study, we will define falls as "unexpected event in which the participants come to rest on the ground, floor, or other lower level" [61,62]. An independent researcher will be in charge of auditing all nursing and medical records to record all falls in the participants over the study period.

## Discussion

Up to April 2009, 58 nonagenarians living in the aforementioned geriatric nursing home meet all the eligibility criteria. Forty-eight of them originally gave their written permission to participate in the study in the previous months. Of these 48 persons, six who were allocated to the control group finally refrained from participating in the study. One participant assigned to the training group had to drop-out from the program after having completed only one training session due to eye surgery. The number of participants who are currently participating in the study within the control and intervention group is n = 12 (2 men and 10 women, mean (SD) age: 92 (3) years, range 90 to 97 years) and n = 18 (3 men and 15 women; mean age: 92 (2) years, range 90 to 96 years) respectively. All these 30 participants successfully completed all of baseline evaluations, included all the strength and functional tests. Of the total of 24 training sessions that we programmed for each participant in the training group, the 19 subjects who are currently in this group have performed an average of 10 sessions, with a mean adherence of 87%. Main reasons for missing a training session were mild upper respiratory tract infections, falls (at night, out of training sessions) or simply forgetting. We noted no major health problem (included absence of falls) associated with the baseline strength tests or training sessions, expect mild muscle pain in some participants.

## Conclusion

The increase in life expectancy together with the dramatic decrease in birth rates in industrialized countries calls the attention to health care systems and public health policymakers to focus attention on promoting healthy lifestyle in the highest sector of the population pyramid. Our study attempts to improve functional capacity and QOL of nonagenarians by implementing an individualised aerobic and strength training program in a geriatric residential care. Results from STRONG will help to better understand the potential of regular PA for improving the well being even in persons aged 90 years or over.

## Abbreviations

1RM: one repetition maximum; ACSM: American College of Sports Medicine: ADLs: activities of daily living; AHA: American Heart Association; ANOVA: analysis of variance; BMI: body mass index; FAC: Functional Ambulation Classification; MMSE: Mini-Mental State Examination; MVPA: moderate-to-vigorous physical activity; PA: physical activity; QOL: quality of life; RCT: randomised controlled trial; RPE: rate of perceived exertion; SF-12: Short Form-12 items.

## Competing interests

The authors declare that they have no competing interests.

## Authors' contributions

JAR conceived the study, and participated in its design and coordination; he also helped to draft the manuscript. JRR participated in the study design and drafted the manuscript. NBA participated in the study design and is currently coordinating and supervising all training sessions. MHV participated in the study design and coordination, subject selection and is currently participating in outcome measurements. PGG participated in the study design and subject selection and is currently participating in outcome measurements. MJSI participated in the study design and subject selection and is currently participating in outcome measurements. NBS participated in the study design and is currently participating in outcome measurements. VOS participated in the study design and is currently participating in outcome measurements. NGS participated in the study design and is currently participating in outcome measurements. ABMP participated in the study design and is currently participating in outcome measurements. CG participated in the study design and is currently supervising training sessions. GRR participated in the study design and is currently supervising training sessions. AL participated in the conception of the study, its design and coordination and drafted the manuscript together with JRR. All authors read and approved the final manuscript.

## Pre-publication history

The pre-publication history for this paper can be accessed here:


